# Management of cerebral ventriculoatrial shunt during pulmonary endarterectomy

**DOI:** 10.1016/j.xjtc.2025.06.023

**Published:** 2025-07-03

**Authors:** Matthew Aizpuru, Benjamin D. Elder, Philip J. Spencer, Sahar A. Saddoughi, Hector R. Cajigas, Mauricio A. Villavicencio

**Affiliations:** a Department of Cardiovascular Surgery, Mayo Clinic, Rochester, Minn; b Department of Neurologic Surgery, Mayo Clinic, Rochester, Minn; c Division of Pulmonary and Critical Care, Department of Medicine, Mayo Clinic, Rochester, Minn

**Figure:**
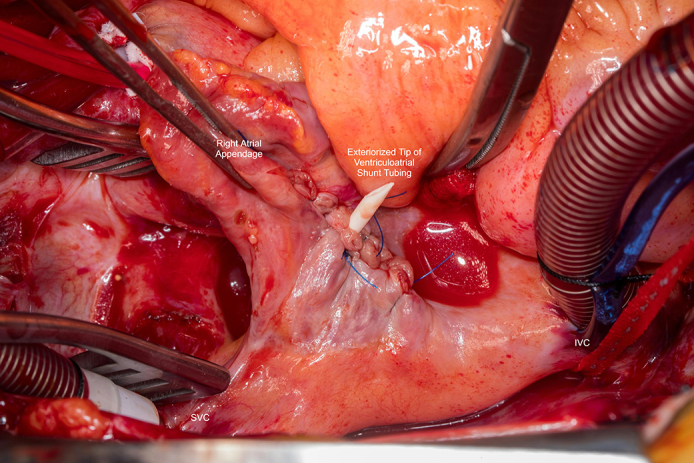
Surgeon's view of the VA shunt tubing exteriorized through the right atrial free wall.

**Central Message**

Management of cerebral ventriculoatrial shunts during cardiopulmonary bypass requires careful planning. Here we describe our management of a VA shunt during pulmonary endarterectomy.

## Case Report

Ventriculoatrial (VA) shunts are an option for management of hydrocephalus when the peritoneal cavity is not suitable. Previously reported complications associated with VA shunts include endocarditis, catheter fracture/migration, and intracardiac thrombosis.^1^^,^^2^ Optimal management of VA shunts during cardiopulmonary bypass is unclear. Here we describe our management of this complex situation with the express written consent of the patient; institutional review board approval was not required.

**Figure 1:**
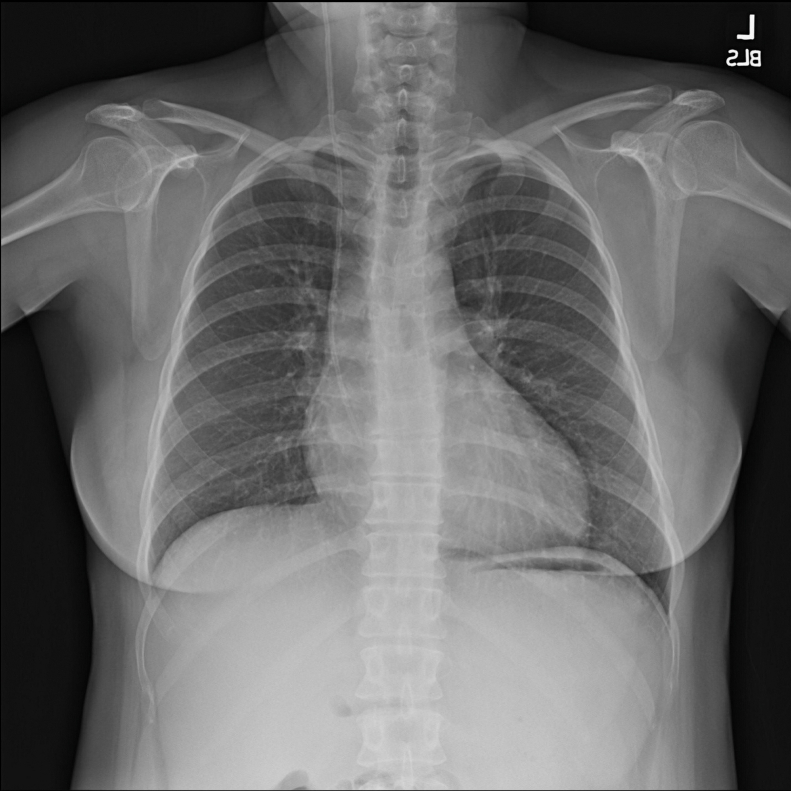
Chest radiograph demonstrating ventriculoatrial shunt tubing terminating in the right atrium.

This is the case of a 40-year-old female patient with history of a VA shunt implanted in childhood, who presented with chronic thromboembolic pulmonary hypertension. Associations between chronic indwelling vascular devices and chronic thromboembolic pulmonary hypertension have been described.^3^ The patient underwent right heart catheterization, which revealed pulmonary arterial pressure of 62/25 (38) mm Hg, pulmonary vascular resistance of 18.6 WU, and a cardiac index of 1.71 L/min/m.^2^ The echocardiogram demonstrated moderate right ventricular dysfunction, and a chest radiograph revealed the VA shunt tip terminating in the right atrium (Figure 1). The VA shunt was a nonprogrammable, low-pressure, pressure-differential valve, originally placed in 1985. She has had multiple revisions of the distal atrial catheter, most recently in 1998. We were concerned that the negative distal pressure associated with vacuum-assist cardiopulmonary bypass would result in a significant pressure differential across the shunt system in the absence of an antisiphon device, and therefore severe overdrainage of cerebrospinal fluid (CSF) and neurologic consequences. We therefore planned for pulmonary endarterectomy with exteriorization of the shunt tip and drainage of CSF into the pericardial well while on cardiopulmonary bypass.

**Figure 2:**
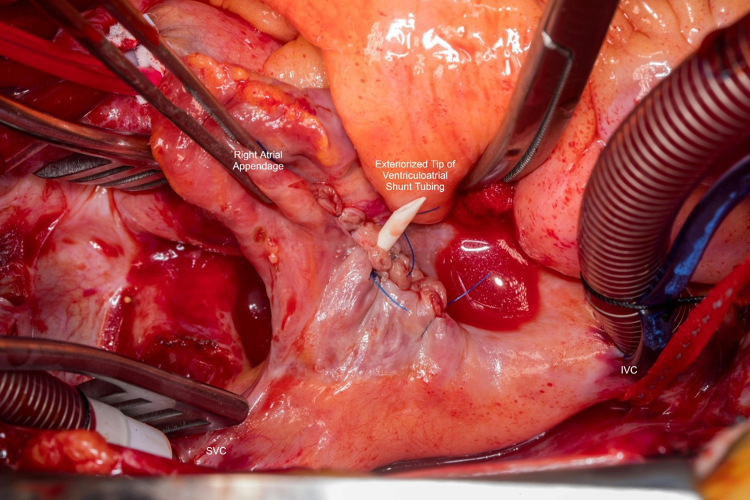
Surgeon's view of the right atrium. The ventriculoarterial shunt tubing has been exteriorized through the free wall of the right atrium. *SVC*, Superior vena cava.

**Figure 3:**
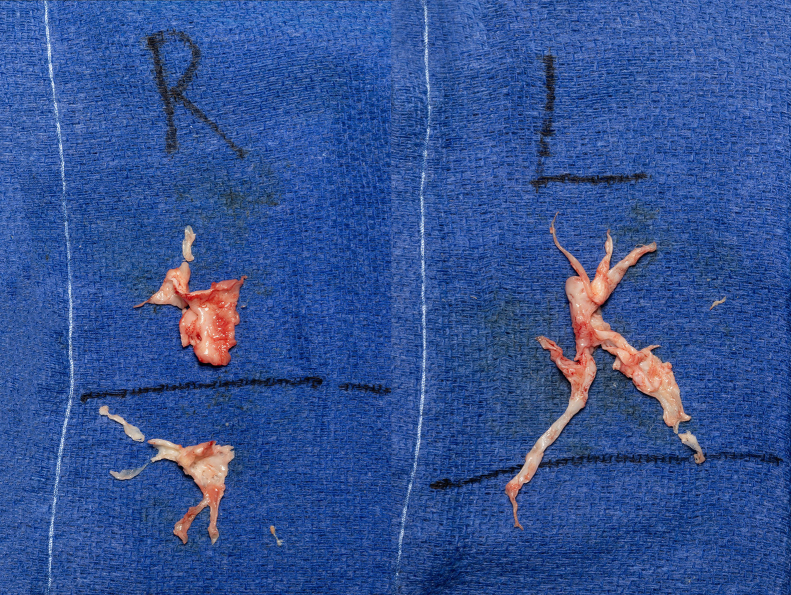
Intraoperative specimen photographs. *R*, Right; *L*, left.

The patient was brought to the operating room, underwent median sternotomy, distal aortic arterial and bicaval venous cannulation with snares. After initiating cardiopulmonary bypass without vacuum-assist, the right atrium was opened and the shunt tip was exteriorized. The atriotomy was closed with 4-0 Prolene (Ethicon) around the shunt tubing, allowing for free flow of the CSF into the pericardial well (Figure 2). The pulmonary endarterectomy was then completed using vacuum-assist cardiopulmonary bypass and deep hypothermic circulatory arrest, 26 minutes on the right side and 16 on the left (Figure 3). The shunt tip was returned to the right atrium at completion of the case. Pulmonary artery pressures normalized in the operating room. The patient recovered well and was discharged from the hospital on the fifth postoperative day without any neurologic sequelae.

## Conflict of Interest Statement

The authors reported no conflicts of interest.

The *Journal* policy requires editors and reviewers to disclose conflicts of interest and to decline handling or reviewing manuscripts for which they may have a conflict of interest. The editors and reviewers of this article have no conflicts of interest.
